# F‐Doped Carbon Nanoparticles‐Based Nucleation Assistance for Fast and Uniform Three‐Dimensional Zn Deposition

**DOI:** 10.1002/advs.202300398

**Published:** 2023-04-17

**Authors:** Lingyun Xiong, Youjoong Kim, Hao Fu, Weiwei Han, Woochul Yang, Guicheng Liu

**Affiliations:** ^1^ Department of Physics Dongguk University Seoul 04620 Republic of Korea; ^2^ School of Energy Power and Mechanical Engineering North China Electric Power University Beijing 102206 China

**Keywords:** dendrite‐free, high charge density, nucleation assistor, three‐dimensional growth, zinc‐metal anodes

## Abstract

Aqueous Zn metal‐based batteries have considerable potential as energy storage system; however, their application is extremely limited by dendrite development and poor reversibility. In this study, to overcome both challenges, F‐doped carbon nanoparticles (FCNPs) are uniformly constructed on substrates (Ti, Zn, Cu, and steel) by a plasma‐assisted surface modification, which endows reversible and uniform deposition of Zn metal. FCNPs with high surface charge density act as nucleation assistors and form numerous homogenous Zn nucleation sites toward Zn 3D growth, which improves Zn plating kinetic and results in uniform Zn deposition. Furthermore, the ZnF_2_ solid electrolyte interface generated during cycling contributes to rapid mass transfer and enhances Zn reversibility, but also suppresses the side reaction. Accordingly, the half‐cell of P‐Ti coupled with Zn exhibits an average Coulombic efficiency of 99.47% with 500 cycles. The symmetric cell of the P‐Zn anode presents a lifespan of over 1500 h at the current density of 5 mA cm^−2^. Notably, the cell works for 100 h at 50 mA cm^−2^. It is believed that this ingenious surface modification broadens revolutionary methods for uniform metallic deposition, as well as the dendrite‐free rechargeable batteries system.

## Graphical abstract

A nucleation assistor made of F‐doped carbon nanoparticles with high surface charge density is proposed on the metal substrate (Zn, Ti, Cu, and steel) for fast and uniform 3D growth of Zn deposition to increase the lifespan for aqueous Zn‐ion rechargeable batteries.

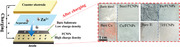

## 1 Introduction

Aqueous Zn‐ion rechargeable batteries (AZIBs) are considered advantageous for large‐format development over organic electrolyte batteries,^[^ ^1^, ^2^, ^3^, ^4^ ^]^ such as lithium‐ion batteries^[^ ^5^, ^6^, ^7^, ^8^ ^]^ and sodium‐ion batteries,^[^ ^9^, ^10^ ^]^ because it is cost‐efficient and environmentally friendly. However, the lifetime of AZIBs is restricted by its low Coulombic efficiency and compromised Zn reversibility during plating and stripping from water consumption.^[^ ^11^, ^12^ ^]^ The dendrite growth through nonuniform electrodeposition is also a significant barrier to application because the growth results in short‐circuiting and reduces the lifespan of the battery.^[^ ^13^, ^14^ ^]^

It had been known that the intrinsic defects and tiny protrusions of the substrate possess a high current density and induce the aggregated Zn deposition on these tips to form a dendrite during the plating process.^[^ ^15^ ^]^ To suppress Zn dendrite growth, numerous efforts have been made to uniformly deposit Zn; they involve controlling Zn^2+^ flux through a protective layer,^[^ ^16^ ^]^ such as TiO_2_ passivation,^[^ ^17^ ^]^ ZnS,^[^ ^18^ ^]^ Zn_3_(PO_4_)_2_,^[^ ^19^ ^]^ ZnF_2_,^[^ ^20^ ^]^ and polymeric layer.^[^ ^21^ ^]^ Besides, the unique characteristics of the polymeric layer coatings playing natural water/O_2_ resistant properties separate the Zn surface from the bulk electrolyte to enhance the shelf life and Zn utilization efficiency. This strategy is similar to the solid electrolyte interface (SEI) in situ protection role, which makes it an attractive and rational alternative to Zn surface modification area.^[^ ^20^ ^]^ However, those protection methods make it difficult for the Zn surface to interact with the electrolyte and increase the Zn^2+^ migration barrier, leading to a higher overpotential during the strapping/platting process.^[^ ^22^, ^23^, ^24^, ^25^ ^]^

**Scheme 1:**
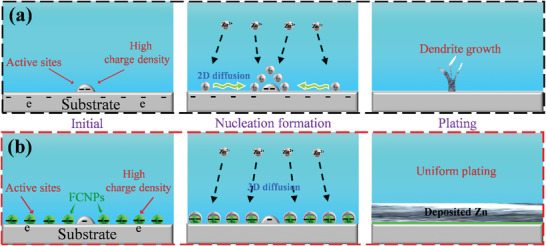
Schematic illustration of the behavior for Zn plating on the a) bare and b) CF_4_ plasma‐treated metal substrate.

The growth behavior of Zn metal is a worthy research direction to achieve uniform Zn plating, particularly for nucleation formation, which significantly affects the metallic Zn growth. The conventional Zn metal electrodeposition on the substrate is schematically illustrated in **Scheme** **1**a. There are a few active sites with a high surface charge density because of defects and protrusions, which would ideally become the nucleation sites during the initial plating process.^[^ ^26^, ^27^, ^28^ ^]^ And then, a larger number of Zn‐ions on the surface diffuse to the favorable sites via 2D diffusion and get the charge for reduction. The ions aggregate on those sites and produce inhomogeneous and agglomerated Zn clusters (or islands) with 2D growth, which results in the formation of dendrites.^[^ ^29^ ^]^

Inspired by this, we proposed that F‐doped carbon nanoparticles (FCNPs) with high surface charge density uniformly assembled on the metal substrates through plasma‐assisted surface modification. The inducement layer of FCNPs with high charge density as Zn nucleation assistors, not only effectively promotes abundant homogenous Zn nucleation devoting to Zn 3D growth, but also processes superior Zn deposition kinetics (Scheme 1b), which is validated by electrostatic force microscope (EFM), linear sweep voltammetry (LSV), chronopotentiometry, chronoamperometry, and nucleation investigation. Furthermore, the ZnF_2_ SEI layer generated during cycling can efficiently suppress the side reaction and improve Zn reversibility. Moreover, this concept is also verified on the Zn, Ti, Cu, and steel substrates with a uniform Zn deposition and a lower nucleation formation overpotential, providing novel insights for uniform Zn deposition.

## 2 Results and Discussion

**Figure 1:**
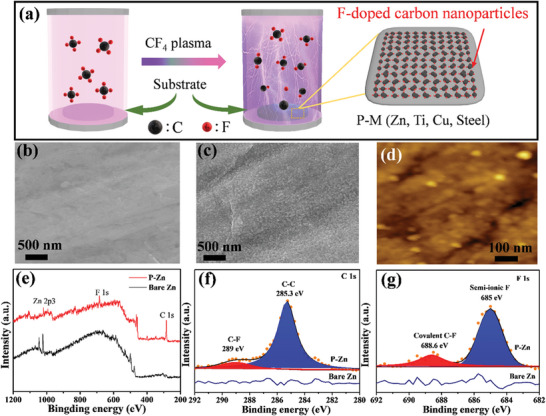
a) Schematic illustration of preparing P‐M electrode. The surface morphology of b) bare Zn and c) P‐Zn. d) AFM of P‐Zn. XPS analysis of bare Zn and P‐Zn electrodes for e) survey spectrum, f) C 1s, and g) F 1s.

**Figure** **1**a illustrates the surface modification of metal electrodes by CF_4_ plasma treatment to synthesize P‐M electrodes. The FCNPs can be generated from the CF_4_ gas through high‐energy plasma ionization.^[^ ^30^, ^31^ ^]^ The morphology of bare and plasma‐treated Zn substrate was examined using SEM measurements. Bare Zn and P‐Zn exhibit a flat and smooth surface, as shown in Figures 1b and 1c, respectively. After CF_4_ plasma treatment for 30 min, an abundance of homogeneous nanoparticles were observed on the surface of the P‐Zn electrode (Figure 1c). The size of these nanoparticles is about ≈30 nm which was measured by the atomic force microscope (Figure 1d). Compared to Zn substrate treated with plasma for 10 min (Figure S1, Supporting Information), it can be observed that the surface was not fully covered by the FCNPs and some defects existed because the number of FCNPs generated was smaller due to the short treatment time. This defect has a negative impact on the Zn electrodeposition, particularly on dendrite production, during the charging and discharging process.^[^ ^30^ ^]^ The morphology of P‐Ti surfaces was also observed FCNPs compared with bare Ti (Figure S2, Supporting Information).

X‐ray photoelectron spectroscopy (XPS) measurements were performed to investigate the chemical composition of P‐Zn. The atomic proportion of C, Zn, and F elements is 81.9 %, 9.4 %, and 8.7 %, respectively (Table S1, Supporting Information). The survey spectrum of P‐Zn shows high intensity of C 1s and F 1s peaks (Figure 1e). Similar results are also reflected on the P‐Ti electrode, in which the atomic proportion of C, Ti, and F elements is 60.9 %, 21.7 %, and 17.4 %, respectively. The high resolution of C 1s is given in Figure 1f, which can be devolved into the C—C and C—F bonds with the binding energies of 285.3 and 289.0 eV,^[^ ^32^ ^]^ respectively, in which the intensity of the C—C peak is significantly higher than the C—F peak. Additionally, the Raman spectra of P‐Zn display obvious D and G peaks at 1336.8 and 1599.3 cm^−1^,^[^ ^33^ ^]^ respectively (Figure S3, Supporting Information). Meanwhile, the covalent F and semi‐ionic F are confirmed by the XPS spectra of F 1s (Figure 1e) with the binding energy of 688.6 and 685.0 eV, respectively, in which a large amount of semi‐ionic F exists.^[^ ^34^, ^35^ ^]^ It is worth noting that the semi‐ionic F can form a SEI layer of ZnF_2_ on the deposited Zn surface, which contributes to uniform Zn deposition and Zn reversibility during cycling, as exhibited (further explanation below). This result is also reflected in P‐Ti (Figure S4, Supporting Information). Contact angle measurement was performed to evaluate the surface wettability of bare Zn, P‐Zn‐10, and P‐Zn electrodes toward the aqueous electrolyte. The bare Zn substrate displays a smaller contact angle of 85° (Figure S5a, Supporting Information). In contrast, after plasma treatment for 10 min (Figure S5b, Supporting Information), the contact angle increases to 120°. As the treatment time increased to 30 min, the contact angle further increases up to 135° (Figure S5c, Supporting Information). This superior non‐wettability is created by the carbon feature of the FCNPs, which can effectively separate the water molecules from the electrode surface to inhibit HER in the plating process which significantly improves charging/discharging efficiency.^[^ ^36^ ^]^ Moreover, various metal substrates (Zn, Ti, Cu, and Steel) exhibit dark color after plasma treatment (Figure S6, Supporting Information). These results prove that the proposed method is a universal approach to successfully fabricate FCNPs on various types of metal substrates through CF_4_ plasma treatment.

**Figure 2:**
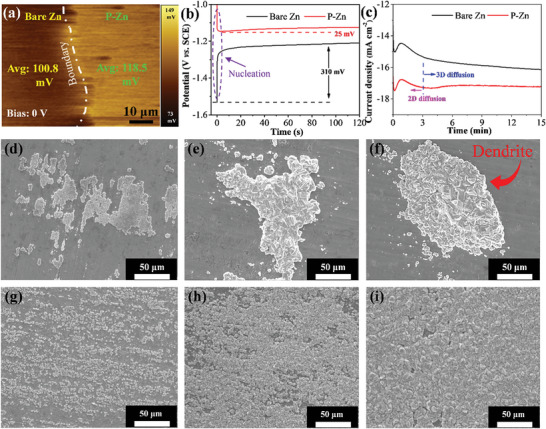
a) EFM, b) Chronopotentiometry, and c) chronoamperometry analysis of bare Zn and P‐Zn electrodes. The SEM images of nucleation formation for d–f) bare Zn and g–i) P‐Zn were deposited for d,g) 2 min, e,f) 5 min, and f,i) 10 min at the current density of 5 mA cm^−2^ in 2 m ZnSO_4_ electrolyte.

The EFM measurements were performed to investigate the surface potential and charge density of bare Zn/P‐Zn. The average surface potential value of P‐Zn (118.5 mV) is 17.7 mV higher than bare Zn (100.8 mV) at a bias of 0 V (**Figure** **2**a), indicating that an abundant charge distribution exists on the P‐Zn surface. Decreasing the bias to −1 V, the differential value is expanded to 38.4 mV (Figure S7, Supporting Information), demonstrating that the P‐Zn surface obtains a higher charge density than bare Zn. At a bias of +1 V, the value decreases to 7.1 mV, showing a similar charge density (bare Ti/P‐Zn is shown in Figure S8, Supporting Information). A higher charge density will facilitate Zn nucleation formation at the initial deposition. According to the chronopotentiometry curves, the nucleation formation overpotential of bare Zn is 310 mV (Figure 2b). In contrast, on the P‐Zn electrode, a small nucleation overpotential occurred due to the nucleation assistor roles of FCNPs, which indicates that a lower nucleation formation energy was required for the abundant homogeneous Zn nucleation generated during this process. Similar behavior is also observed in bare Ti/P‐Ti, bare steel/P‐steel, and bare Cu/P‐Cu electrodes (Figure S9, Supporting Information). Additionally, to further scrutinize the role played by FCNPs to promote the Zn growth mechanism, chronoamperometry was performed at an overpotential of −150 mV (Figure 2c). For bare Zn, the current density is gradually increased for 15 min, which indicates that long and intense 2D diffusion occurred. The absorbed Zn‐ions on the surface diffuse to the favorable sites and obtain the charge for the reduction. The ions then aggregate on these sites and grow into dendrites.^[^ ^3^ ^]^ For the P‐Zn electrode, the 2D diffusion process occurs within 3 min and achieves a higher current density, suggesting that abundant Zn nucleation is generated due to the nucleation assistor roles of FCNPs. Furthermore, constant and stable 3D diffusion proceeds for 12 min with a current density of 17.2 mA cm^−2^, which indicates that the absorbed Zn‐ion on the surface is directly reduced to Zn metal without 2D diffusion. With the constant 3D Zn deposition, the nucleation seeds grow and come into contact together to form a uniform and high‐density Zn deposited layer.

To directly evaluate the nucleation assistor function of the FCNPs contributing to Zn growth behavior, Figure 2d–i shows the SEM images of bare and P‐Zn electrode surface for Zn electrodeposition of 2, 5, and 10 min at a current density of 5 mA cm^−2^. The bare Zn grown for 2 min exhibits aggregated irregularly shaped and sized Zn nucleation islands (Figure 2d). Continuous plating for 5 min leads to coalescence growth of smaller islands into larger islands (Figure 2e). After deposition for 10 min, vertical growth of the Zn islands becomes dominant and results in dendrite formation (Figure 2f). In comparison, the P‐Zn growth for 2 min presents nucleation and distribution of Zn islands with relatively homogeneous and smaller sizes (Figure 2g). As the growth time increases to 10 min, the islands prefer to grow laterally larger and cover the whole surface (Figure 2h,i). The results indicate that FCNPs can lead to the homogeneous growth of the Zn layer on the Zn substrate during plating. In addition, this strategy was also validated on various substrates (Zn, Ti, Cu, and steel), as shown in Figure S10, Supporting Information. After plating on the partially plasma‐treated metal surfaces, the optical images of bare Zn/P‐Zn, bare Ti/P‐Ti, bare steel/P‐steel, and bare Cu/P‐Cu show that a large amount of Zn layer is uniformly grown on the plasma‐treated area, whereas the bare substrate achieved seldom and irregular Zn growth. Therefore, the FCNPs‐modified substrate presents outstanding uniform plating for Zn.

**Figure 3:**
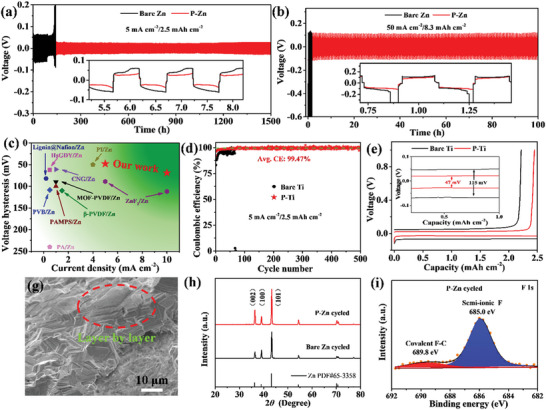
Long‐cycle performance of symmetric cells using P‐Zn and bare Zn electrodes at a) 5 mA cm^−2^ and b) 50 mA cm^−2^, and the corresponding galvanostatic voltage curves are inserted. c) Comparison of electrochemical performance of reported works. d) Coulombic efficiency of bare Ti and P‐Ti coupled with Zn foil for cycling performance at 5 mA cm^−2^, and e) the corresponding galvanostatic voltage curves. f) SEM morphology, g) XRD, and h) XPS of F 1s of P‐Zn electrode after 50 cycles at a current density of 5 mA cm^−2^.

The effect of FCNPs on the lifespan of Zn plating and strapping was investigated by symmetric cells (P‐Zn//P‐Zn) and half‐cells (Zn//P‐Ti). As shown in **Figure** **3**a, the P‐Zn electrode conveys an ultralong cycling life of 1500 h at a current density of 5 mA cm^−2^ with a capacity of 2.5 mAh cm^−2^, while the bare Zn electrode is dendritic and shorts out after 90 h. The corresponding voltage hysteresis of P‐Zn (≈25 mV) is markedly smaller than that of bare Zn (≈60 mV) (inset of Figure 3a). Applying a current density of 10 mA cm^−2^ with a capacity of 5 mAh cm^−2^, the cycling life of bare Zn, P‐Zn‐10, P‐Zn, and P‐Zn‐60 is 58, 101, 260, and 74 h, and the corresponding voltage hysteresis is ≈51, ≈27, ≈29, and ≈31 mV, respectively (Figure S11, Supporting Information). The P‐Zn electrode possesses durable cycling performance and lower voltage hysteresis. Surprisingly, the P‐Zn electrode also obtains proper operation for 100 h with a voltage hysteresis of ≈100 mV under a considerably high current density of 50 mA cm^−2^ with a large plating/strapping capacity of 8.3 mAh cm^−2^ (Figure 3b). In sharp contrast, the bare Zn died by the dendritic formation at the first discharging process and could not be cycled. Furthermore, compared with recent literature, the P‐Zn delivers outstanding durability and lifespan (Figure 3c). Additionally, the Coulombic efficiency is a useful index parameter to evaluate the reversibility of Zn during the plating/stripping process. Figure 3d shows that the Zn//P‐Ti half‐cell delivers a 500 cycles life with an outstanding average Coulombic efficiency of 99.47%. When applying bare Ti as the counter electrode, the cell dies after 65 cycles with an average Coulombic efficiency of 94.5%. Meanwhile, the Zn//P‐Ti harvests a voltage hysteresis of 47 mV much lower than that of Zn//Ti (115 mV) as shown in Figure 3e. The plasma‐treated Cu electrode coupled with Zn foil also delivers outstanding performance compared with bare Cu. The Zn//P‐Cu half‐cell obtains a longer cycling life of 114 cycles and a higher average Coulombic efficiency of 99.43% than those of Zn//Cu (74 cycles and 99.25%) (Figure S12a, Supporting Information). Meanwhile, the voltage hysteresis of Zn//P‐Cu is 25.6 mV which is lower than that of Zn//Cu (51.6 mV) (Figure S12b, Supporting Information). The excellent cycling life, outstanding Coulombic efficiency, and lower voltage hysteresis of plasma‐treated electrodes exhibit dendrite‐free, high reversibility, and fast deposition kinetics of Zn.

Furthermore, the plating kinetics were investigated by LSV measurements for bare Zn and P‐Zn electrodes in ZnSO_4_ and Na_2_SO_4_ electrolytes. For the ZnSO_4_ electrolyte, the overpotential of P‐Zn is −1.13 V at a current density of 10 mA cm^−2^, which is lower than that of the bare Zn electrode (−1.16 V) (Figure S13a, Supporting Information), implying that the P‐Zn electrode possesses faster deposition kinetics. This process includes Zn reduction and HER. To eliminate the influence of the HER side reaction, the LSV measurement was performed in 1 m Na_2_SO_4_ electrolyte. P‐Zn gets an overpotential of −2.1 V, which is markedly higher than that of bare Zn (−1.8 V) (Figure S13b, Supporting Information), indicating that HER reaction is suppressed on the P‐Zn electrode because of the water‐blocking function via a hydrophobic property of FCNPs. Moreover, electrochemical impedance spectroscopy and the fitting results show that P‐Zn obtains a dramatically lower charge transfer impendence (*R*_ct_) of 90.3 Ω than that of bare Zn (469.3 Ω) (Figure S14 and Table S2, Supporting Information). The lower *R*_ct_ contributes to the rapid mass transfer between the electrode surface and the electrolyte which contributes to the fast Zn plating kinetics.^[^ ^37^ ^]^ Furthermore, the cyclic voltammetry (CV) curves of P‐Ti exhibit high oxidation and reduction current density than that of bare Ti, which also reveals that FCNPs‐modified Ti achieves high Zn redox kinetics (Figure S15, Supporting Information). These results declare that the plasma‐treated metal substrates possess high Zn deposition kinetics and this behavior is attributed to the synergistic effect of high charge distribution and nucleation assistor role of the FCNPs.

To understand the enhanced performance mechanism, the P‐Zn electrode and bare Zn were dissected after 50 cycles. The side‐section morphology of P‐Zn displays a flat, dense, and smooth surface and Zn growth layer by layer (Figure 3f) which can be attributed to the inducement of FCNPs for homogeneous Zn plating. For the bare Zn electrode, the surface is a distinct cluster of porous dendrites (Figure S16, Supporting Information). The corresponding 3D height image of the P‐Zn electrode presents a notably regular surface with an average roughness of 6.38 µm (Figure S17a, Supporting Information). In comparison, bare Zn displays a vitally rough surface with an average roughness of 40.84 µm (Figure S17b, Supporting Information). Interestingly, the XRD patterns show that the (002) lattice plane of P‐Zn is obversely higher than that of bare Zn after 50 cycles (Figure 3g). The (002) plane of Zn crystals is grown layer by layer, as reflected in Figure 3g, and endows uniform and dendrite‐free electrodes to achieve long‐cycling plating and stripping performance.^[^ ^38^ ^]^

The XPS analysis of the P‐Zn electrode after 50 cycles shows that the F elemental proportion is 6.2% (Table S1, Supporting Information), most of which is ionic F (Figure 3h), indicating an abundance ZnF_2_ formed on the surface. The top‐layer of the ZnF_2_ forms SEI, which can efficiently suppress the HER and improve Zn reversibility.^[^ ^39^ ^]^ Strangely, the C atom rapidly decreases to 2.0 % (Table S2, Supporting Information) and the Raman spectra of the D and G peaks disappear after 50 cycles (Figure S18, Supporting Information), demonstrating that a large amount of C atom vanishes from the surface after cycling. However, the majority of the C element was observed underneath the deposited Zn in the cross section of the EDS results (Figure S19, Supporting Information). The carbon found underneath the deposited Zn is attributed to the FCNPs serving as nucleation assistor functions on the bottom and the Zn metal deposited above it. Therefore, the sandwich structure of the cycled P‐Zn electrode (Figure S20, Supporting Information) presents some unique advantages: the top‐layer protector ZnF_2_ can efficiently inhibit the HER and self‐corrosion side reactions and improves Zn reversibility; the bottom‐layer of FCNPs provides nucleation assistance, fast kinetics, and uniform Zn deposition; the active inner‐Zn metal is protected by the top ZnF_2_ layer and induced by the FCNPs bottom‐layer to achieve high reversibility, excellent redox ability, and dendrite‐free.

**Figure 4:**
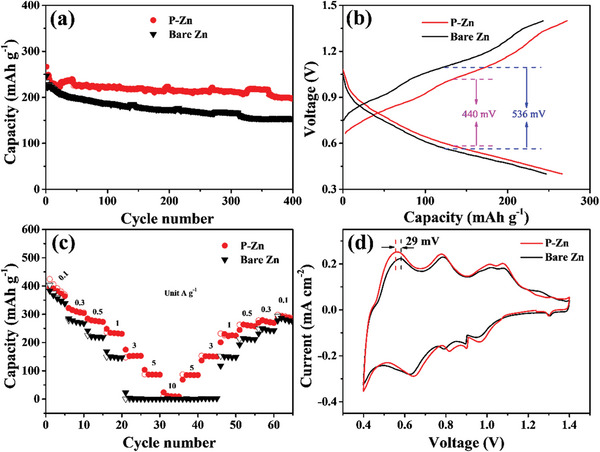
The full batteries performance test of bare Zn//NH_4_V_4_O_10_ and P‐Zn//NH_4_V_4_O_10_ cells. a) Long‐cycling performance, b) voltage–capacity profiles for the first cycles, c) rate performance, and d) CV curves at the scan rate of 0.1 mV s^−1^.

To evaluate the application potential of P‐Zn‐based full batteries, NH_4_V_4_O_10_ was used as a cathode electrode and coupled with bare Zn and P‐Zn electrodes. The long‐cycling performance at 0.5 A g^−1^ showed that the P‐Zn‐based cell achieves excellent stability and an outstanding reversible capacity of 202 mAh g^−1^ for 400 cycles (**Figure** **4**a). In comparison, the bare Zn‐based cell just displays 152 mAh g^−1^ after 400 cycles, which is far undistinguished from the P‐Zn‐based cell. It is worth noting that the P‐Zn‐based cell obtains a higher capacity of 267.2 mAh g^−1^ with a lower polarization (440 mV) for the charging/discharging process, whereas the bare Zn just maintains 247.4 mAh g^−1^ with a higher polarization voltage (536 mV), as presented in Figure 4b. This phenomenon occurs because the P‐Zn electrode possesses outstanding reversible and redox kinetics of Zn metal. The enhanced electrochemical properties are also reflected by the rate performance (Figure 4c). The P‐Zn‐based cell exhibits 362.7, 307.7, 276.2, 228.5, 154.2, 84.2, and 12.9 mAh g^−1^ at the current density of 0.1, 0.3, 0.5, 1.0, 3.0, 5.0, and 10.0 A g^−1^, respectively. By sharp contrast, the specific capacity of bare Zn is 337.1, 264.1, 217.2, 144.2, 4.7, and 0.7 A g^−1^ at the same current densities, respectively. Particularly, at high current density (3, 5, and 10 A g^−1^), the P‐Zn‐based cells far outperform the bare Zn cell. Additionally, the CV patterns of the two cells possess redox peaks (Figure 4d), which are associated with the insertion/desertion of Zn^2+^ in the cathode of a full battery.^[^ ^40^ ^]^ However, the oxidation peaks of P‐Zn cells shifted to a lower voltage (≈29 mV) and the reduction peak shifted to a high voltage, revealing higher oxidation and reduction kinetics of metallic Zn achieved by the P‐Zn electrode, which is consistent with the outlined results.

## 3 Conclusion

A nucleation assistor of FCNPs with high surface charge density on the metal substrate (Zn, Ti, Cu, and steel) was proposed for fast and uniform 3D growth of Zn deposition to achieve a long lifespan for AZIBs. The FCNPs as Zn nucleation assistors facilitate Zn 3D uniform growth and inhibit dendrite formation. In doing so, the FCNPs obversely avoid the agglomerated nucleation formation by 2D diffusion. More notably, the high surface charge density of the FCNPs interface has the potential to improve Zn deposition kinetics by providing more reaction charges. Moreover, the hydrophobic property of FCNP interfaces can suppress the side reaction of HER by separating water from the electrode surface. Furthermore, the in situ formation of the ZnF_2_ SEI generated during cycling can efficiently suppress HER and improve Zn reversibility. Therefore, the half‐cell of P‐Ti//Zn delivers an ultralong lifespan of 500 cycles with an average Coulombic efficiency of 99.47%. The symmetric cell of the P‐Zn anode presents over 1500 h at the current density of 5 mA cm^−2^. Notably, the cell also works for 100 h at 50 mA cm^−2^. These fundamental findings provide insightful strategies for exploring surface modification toward fast and uniform Zn deposition for low polarization and ultralong lifespan Zn‐ion batteries.

## Conflict of Interest

The authors declare no conflict of interest.

## Supporting information

Supporting Information

## Data Availability

The data that support the findings of this study are available from the corresponding author upon reasonable request.
