# Supplementary material for: F‐Doped Carbon Nanoparticles‐Based Nucleation Assistance for Fast and Uniform Three‐Dimensional Zn Deposition
Source: Adv Sci (Weinh). 2023 Apr 17;10(16):2300398. doi: 10.1002/advs.202300398 (PMC10238181; doi:10.1002/advs.202300398)
Supplement: Supplementary file 1 — Supporting Information [file ADVS-10-2300398-s001.pdf]

ADVANCED SCIENCE
Open Access

Supporting Information

for *Adv. Sci.*, DOI 10.1002/advs.202300398

F-Doped Carbon Nanoparticles-Based Nucleation Assistance for Fast and Uniform Three-Dimensional Zn Deposition

Lingyun Xiong, Youjoong Kim, Hao Fu, Weiwei Han, Woochul Yang and Guicheng Liu**

Supplementary Information for

F-doped carbon nanoparticles based nucleation assistance for fast and uniform 3-dimensional Zn deposition

Lingyun Xiong, Youjoong Kim, Hao Fu, Weiwei Han, Woochul Yang*, Guicheng Liu*

Experimental

Preparation of P-M electrode

Plasma-treated metal substrates (P-M, M: Ti, Zn, steel, and Cu) were prepared in the plasma chamber (FEMTOSCIENCE, CIONE) for 30 min using CF_4 gas. The gas flow velocity was set to 10 sccm, the power density to 90 W, the frequency to 90 kHz, and the metal substrate area to 5 cm × 5 cm.

Battery assembly and electrochemical measurements

The battery performance was systemically demonstrated using P-Zn//P-Zn symmetric cells, Zn//P-Ti half cells, and P-Zn// $NH_4V_4O_{10}$ full cells. All cells were tested at room temperature and assembled into CR2032 coin cells with 80 uL of electrolyte. The constant current charging–discharging and Coulombic efficiency were performed on a LANHE battery test system (CT-3001A, Wuhan, China). The P-Zn//P-Zn symmetric cells were tested at a current density of 5, 10, and 50 mA cm^{-2} with a capacity of 2.5, 5, and 8.3 mAh cm^{-2}, respectively. The half cells were tested at 5 mA cm^{-2} with 2.5 mAh cm^{-2} capacity and the Coulombic efficiency was

evaluated through plating to stripping capacity ratio during each cycle. Cyclic voltammetry (CV), linear sweep voltammetry (LSV), and electrochemical impedance spectroscopy (EIS, frequency range: 0.01–10^6 Hz) were conducted on a Biologic VMP electrochemical station. Specifically, the CV measurements of half cells and full cells were tested at 2 mV s^{-1} and 0.1 mV s^{-1}, respectively. The LSV measurements were conducted at a scan rate of 5 mV s^{-1} in 1 mol L^{-1} Na_2SO_4 and 2 mol L^{-1} $ZnSO_4$ electrolyte for a three-electrode system. The three-electrode system of the working, counter, and reference electrodes were Zn foil, Pt plate, Hg/HgCl (saturated KCl), respectively. To confirm the inducement of FCNPs assisting the Zn nucleation formation and 3D diffusion in the plating process, the chronopotentiometry and chronoamperometry measurements were conducted at a polarization of -5 mA cm^{-2} and a bias of -150 mV in 2 mol L^{-1} $ZnSO_4$ electrolyte for a three-electrode system, respectively.

Synthesis of $NH_4V_4O_{10}$ cathode

Ammonium metavanadate (1.7 g) and oxalic acid dehydrate (2.1 g) were dissolved in 60 mL of deionized water with stirring at 60 ºC for 1 h to form a yellow-green solution. Afterward, the solution was heated in a polytetrafluoroethylene container at 180 ºC for 8 h for the hydrothermal reaction. Then, the $NH_4V_4O_{10}$ precipitate was obtained by washing with ethanol and distilled water several times. Finally, the cathode slurry, consisting of $NH_4V_4O_{10}$, carbon nanotube, polyvinylidene fluoride, and N-methyl-2-pyrrolidone (NMP) with a weight ratio of 6 : 3: 1: 10, was coated on Ti foil to obtain the cathode electrode by drying in an oven for one night at 80 ºC.

Characterization

The morphology images of the electrodes were tested using field emission scanning electron microscopy (FESEM, Hitachi S-4800) with an energy-dispersive X-ray spectroscopy. The X-

ray photoelectron spectroscopy (XPS, Veresprobe II, ULVAC-PHI) measurements defined the chemical composition of electrodes with Al Kα radiation (hv = 1486.6 eV). The surface potential and charge distribution were defined by the electrostatic force microscope (EFM, A. P. E. Research, A100). The Raman characteristics of the PPAM-Zn surface were obtained by Raman spectral (MODEL BX41RF-LED). The contact angle between water and the bare Ti foil and P-Ti foil was measured by a contact angle goniometer (ramé-hart Model 250). The 3D height images were collected using a 3D scanning confocal laser microscopy (Keyence, VK-2000).

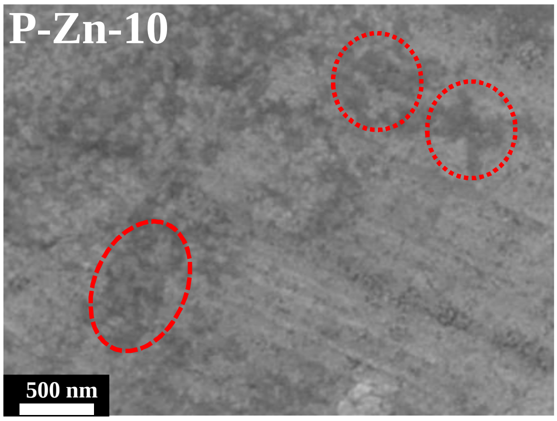

Figure S1. Surface morphology of P-Zn-10.

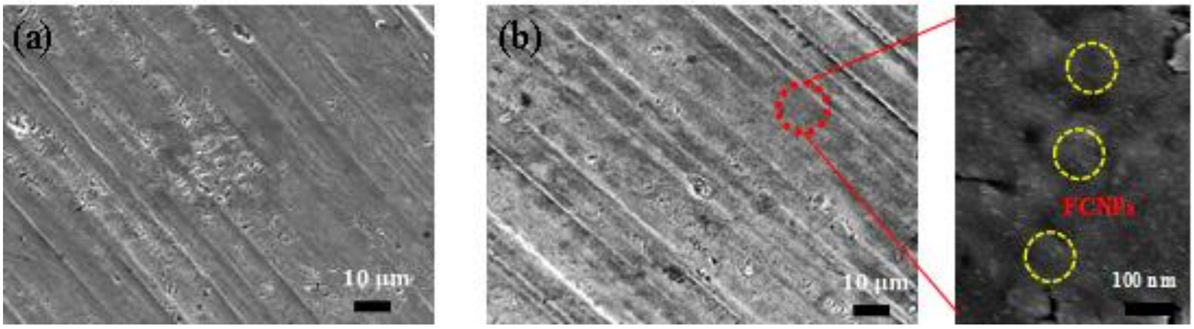

Figure S2. Surface morphology of (a) bare Ti and (b) P-Ti.

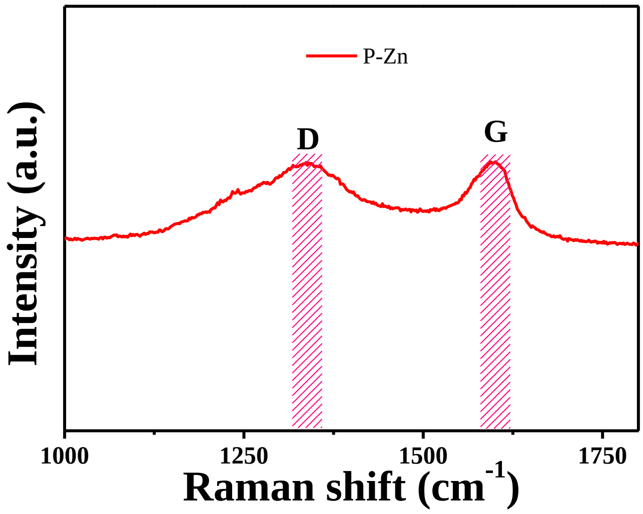

Figure S3. Raman spectral of P-Zn.

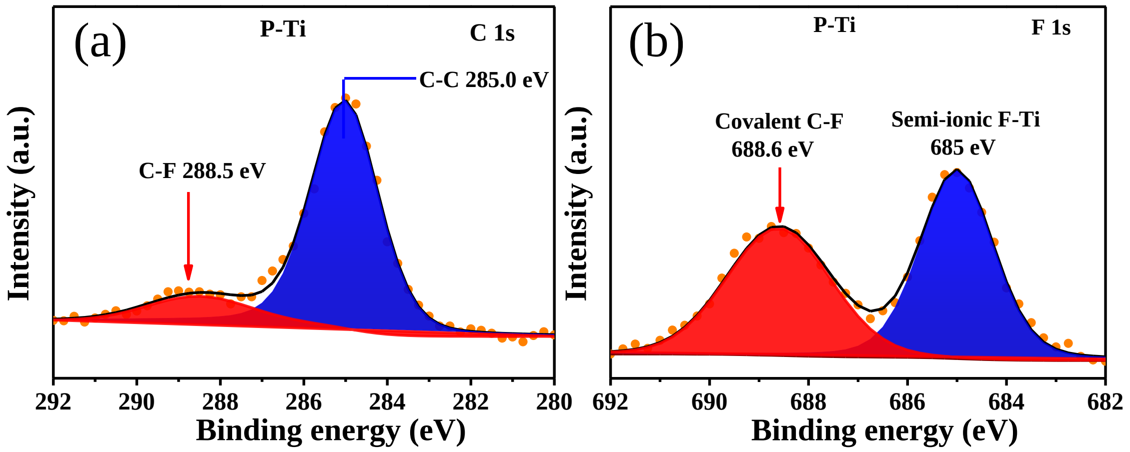

Figure S4. XPS analysis of P-Ti electrode for (a) C 1s and (b) F 1s.

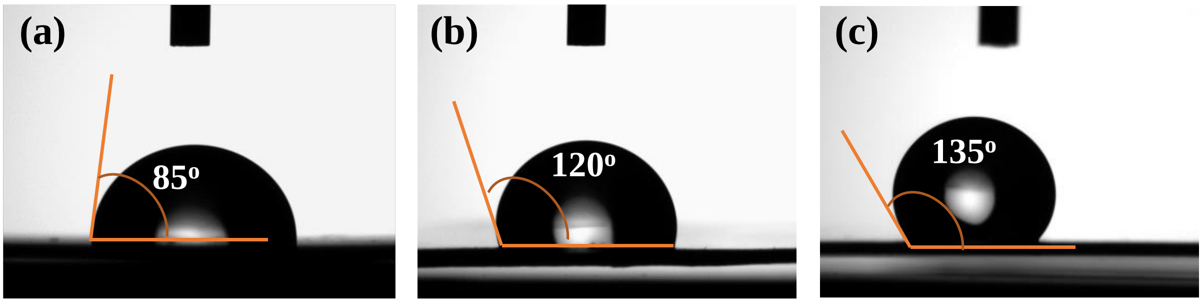

Figure S5. Contact angle of (a) bare Zn, (b) P-Zn-10, and (c) P-Zn.

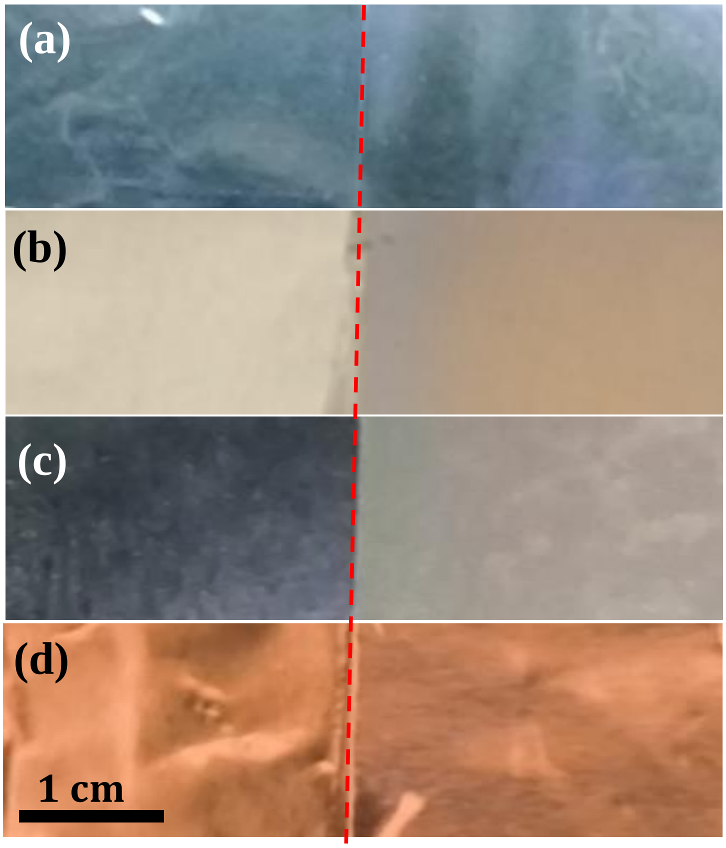

Figure S6. Optical images of bare (left)/plasma-treating (right) electrodes of (a) Zn, (b) Ti, (c) Steel, and (d) Cu substrates.

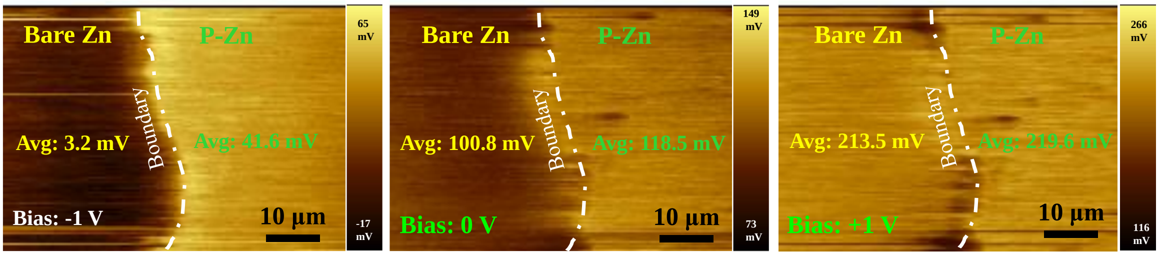

Figure S7. EFM dates of bare Zn/P-Zn at a bias of (a) -1 V, (b) 0 V, and (c) +1 V.

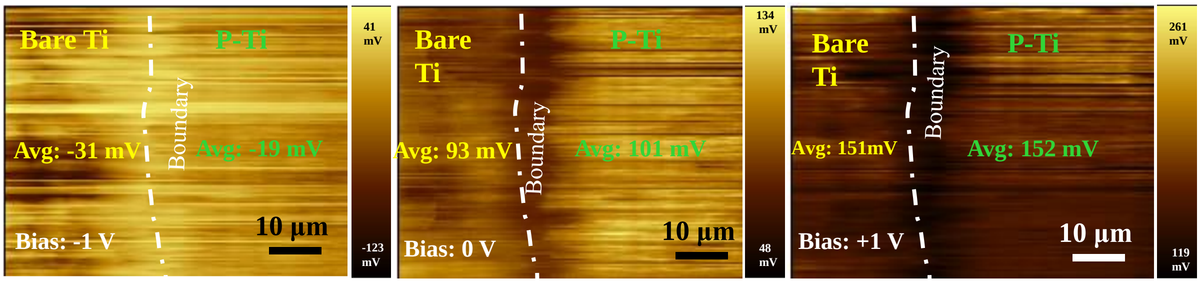

Figure S8. EFM dates of bare Ti/P-Ti at a bias of (a) -1 V, (b) 0 V, and (c) +1 V.

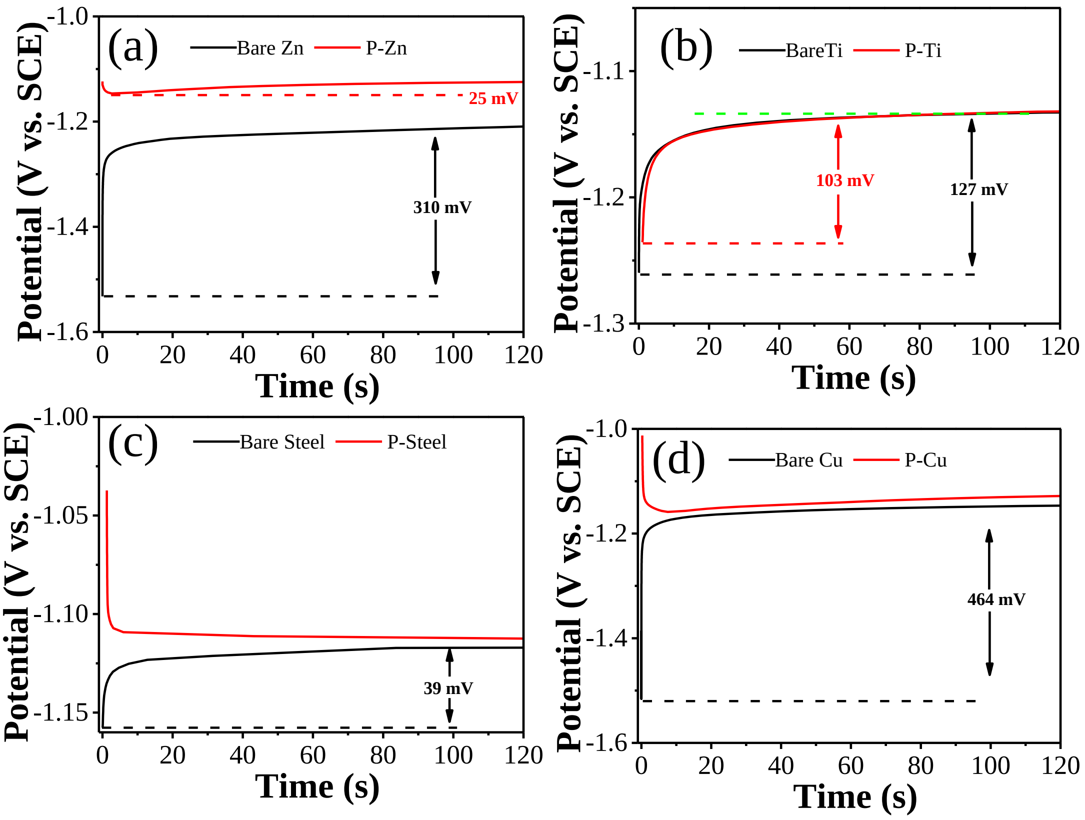

Figure S9. Chronopotentiometry measurement of (a) bare Zn/P-Zn, (b) bare Ti/P-Ti, (c) bare steel/P-steel, and (d) bare Cu/P-Cu.

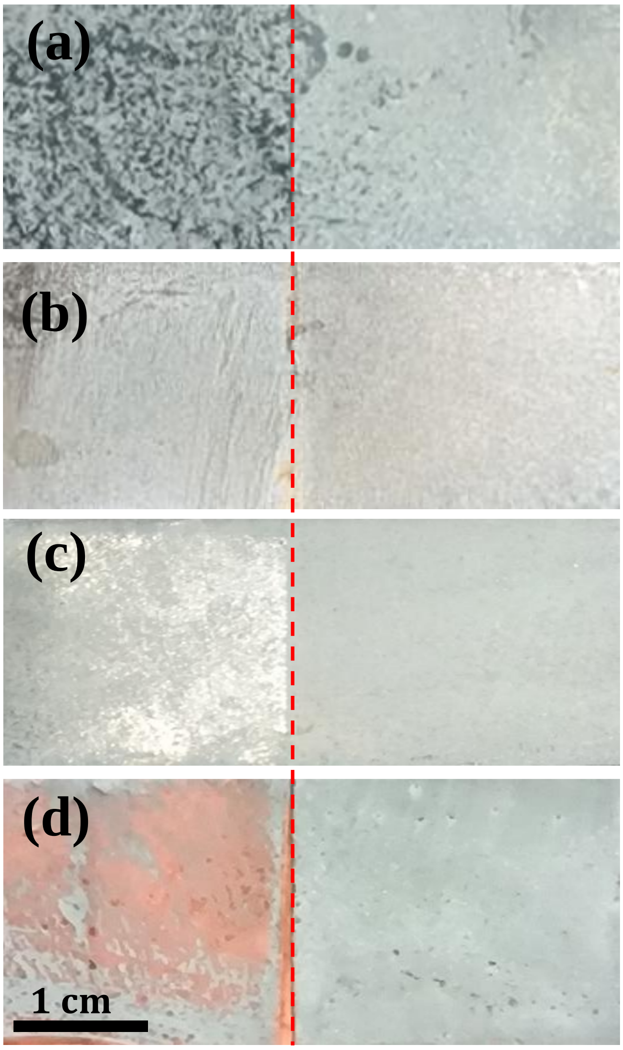

Figure S10. Optical images of bare (left)/plasma-treating (right) electrodes of (a) bare Zn/P-Zn, (b) bare Ti/P-Ti, (c) bare steel/P-steel, and (d) bare Cu/P-Cu after plating.

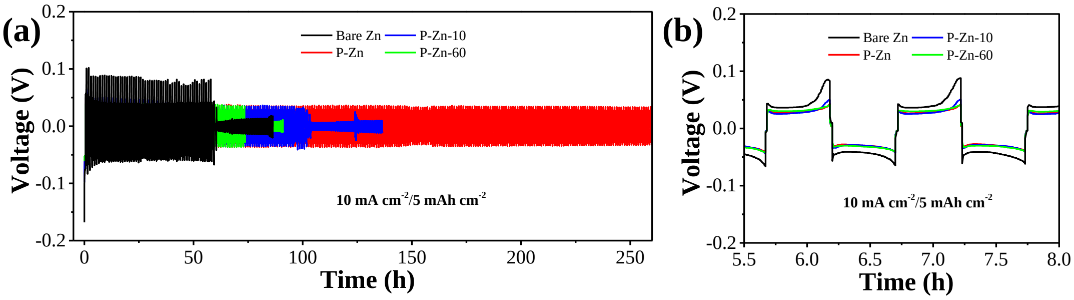

Figure S11. (a) long-cycle performance of symmetric cells for Bare Zn, P-Zn-10, P-Zn, and P-Zn-60 electrodes at 10 mA cm^{-2} (b) corresponding voltage hysteresis.

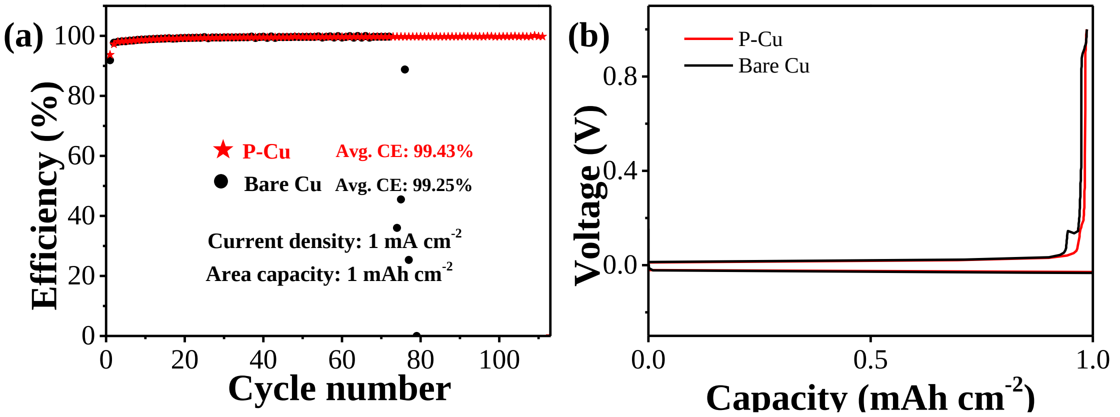

Figure S12. (a) Coulombic efficiency of bare Cu and P-Cu coupled with Zn foil for cycling performance at 1 mA cm^{-2}, and (b) the corresponding galvanostatic voltage curves.

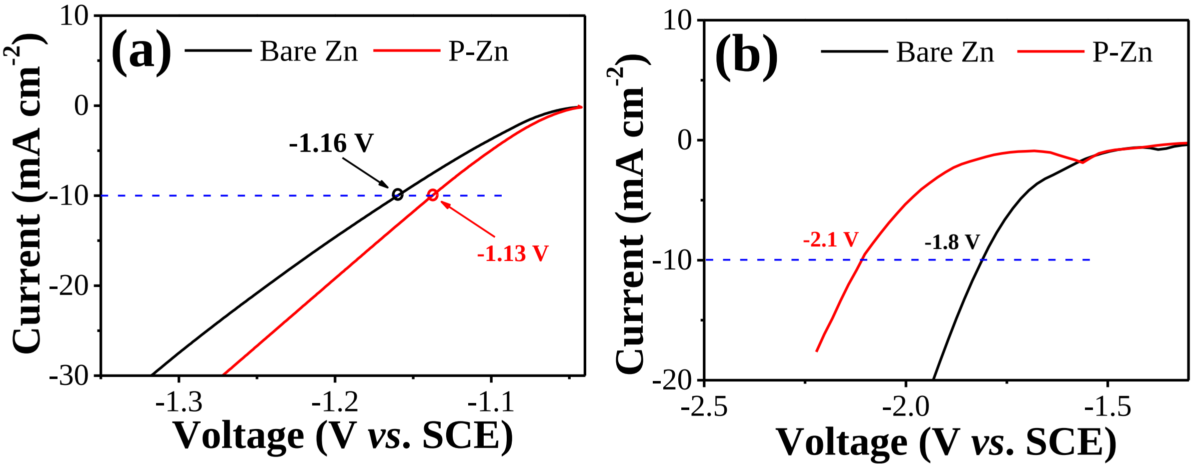

Figure S13. LSV measurement of bare Zn and P-Zn electrodes in (a) 2 M $ZnSO_4$ and (b) 1 M Na_2SO_4 electrolyte.

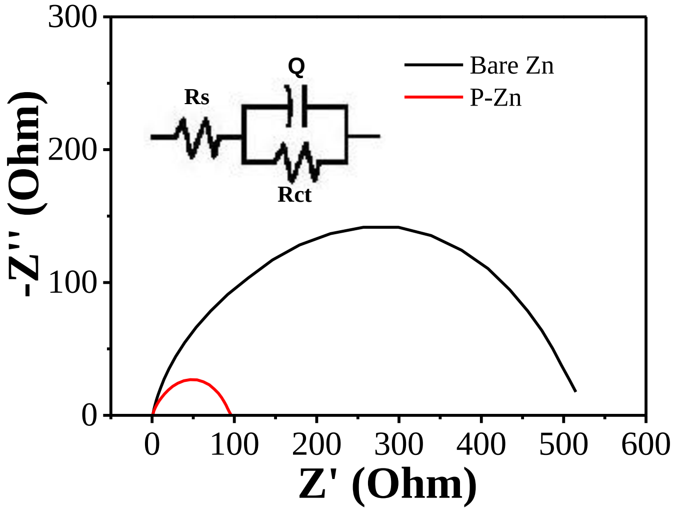

Figure S14. EIS curves of bare Zn and P-Zn. The equivalent fitting diagram is inserted.

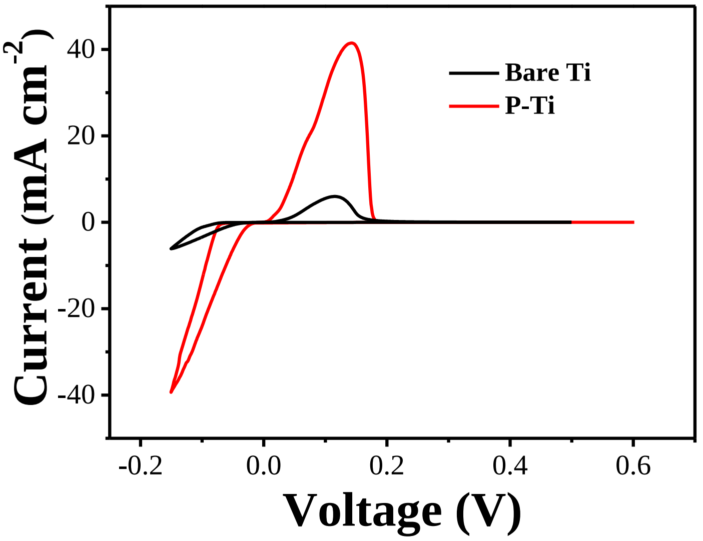

Figure S15. CV curves of bare Ti and P-Ti.

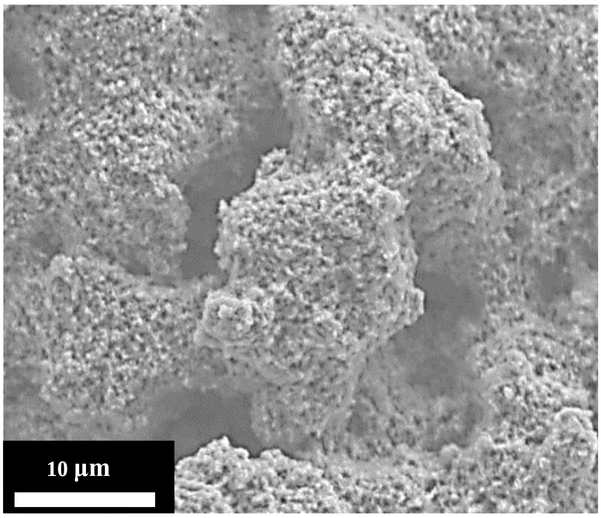

Figure S16. SEM images of bare Zn after 50 cycles

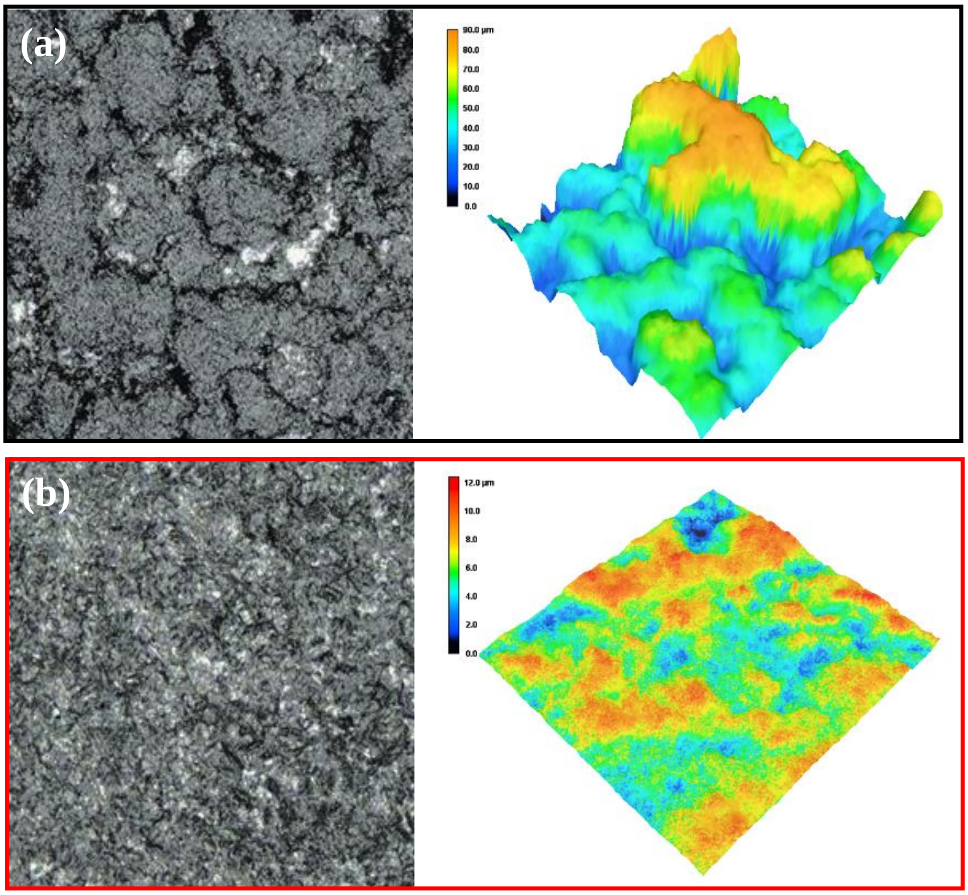

Figure S17. 3D height images of (a) bare Zn and (b) P-Zn after 50 cycles.

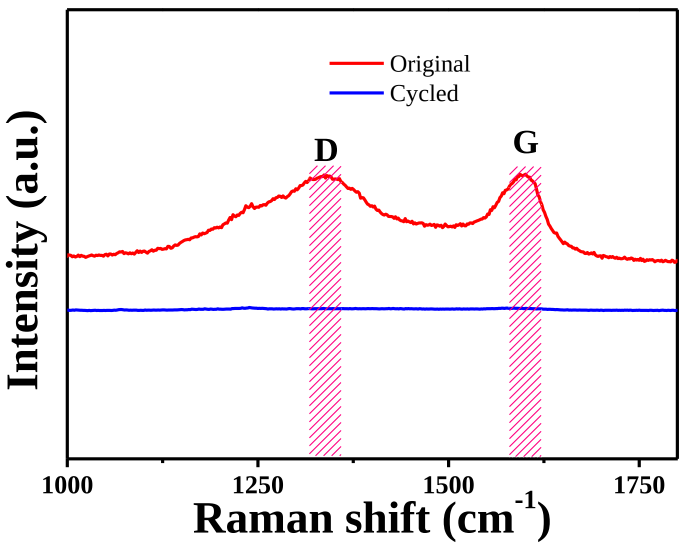

Figure S18. Raman spectra of P-Zn and P-Zn after 50 cycles.

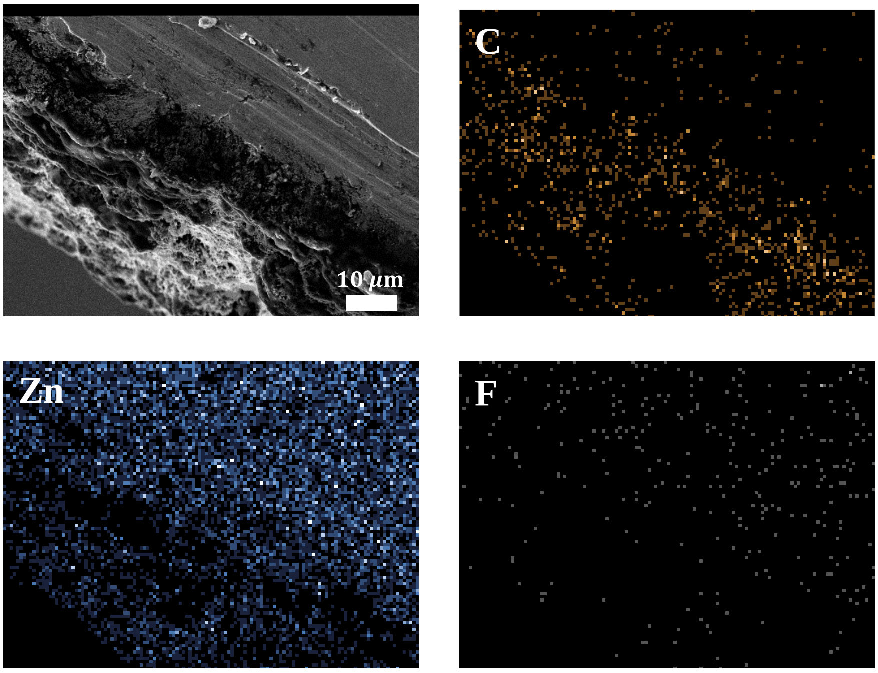

Figure S19. Cross-section EDS mapping of P-Zn after 50 cycles.

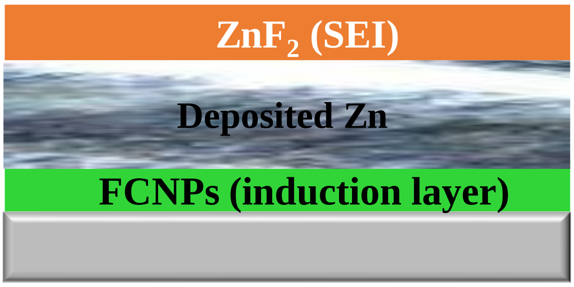

Figure S20. Sandwich structure of cycled P-Zn electrode

Table S1. Elemental composition from XPS measurement.

Element<br>Atomic %	C	F	Zn	Ti
P-Zn	81.9	9.4	8.7	-- --
P-Ti	60.9	21.7		17.4
P-Zn cycled	2.0	6.2	91.8	-- --

Table S2. Fitting results of the alternating-current impedance.

Sample	bare Zn	P-Zn
Rs (Ω)	0.78	0.75
Rct (Ω)	469.3	90.3
